# Aroma profiles of sweet cherry juice fermented by different lactic acid bacteria determined through integrated analysis of electronic nose and gas chromatography–ion mobility spectrometry

**DOI:** 10.3389/fmicb.2023.1113594

**Published:** 2023-01-16

**Authors:** Jun Wang, Bo-Cheng Wei, Xin Wang, Yan Zhang, Yun-Jin Gong

**Affiliations:** ^1^School of Biology, Food and Environment, Hefei University, Hefei, China; ^2^School of Food and Biological Engineering, Hefei University of Technology, Hefei, China

**Keywords:** sweet cherry juice, lactic acid bacteria, electronic nose, gas chromatography–ion mobility spectrometry, volatile compounds

## Abstract

Sweet cherries are popular among consumers, with a recent explosion in sweet cherry production in China. However, the fragility of these fruits poses a challenge for expanding production and transport. With the aim of expanding the product categories of sweet cherries that can bypass these challenges, in this study, we prepared sweet cherry juice fermented by three different lactic acid bacteria (LAB; *Lactobacillus acidophilus*, *Lactobacillus plantarum*, and *Lactobacillus rhamnosus* GG), and evaluated the growth, physiochemical, and aroma characteristics. All three strains exhibited excellent growth potential in the sweet cherry juice; however, *Lactobacillus acidophilus* and *Lactobacillus plantarum* demonstrated more robust acid production capacity and higher microbial viability than *Lactobacillus rhamnosus* GG. Lactic acid was the primary fermentation product, and malic acid was significantly metabolized by LAB, indicating a transition in microbial metabolism from using carbohydrates to organic acids. The aroma profile was identified through integrated analysis of electronic nose (E-nose) and headspace gas chromatography–ion mobility spectrometry (HS-GC–IMS) data. A total of 50 volatile compounds characterized the aromatic profiles of the fermented juices by HS-GC–IMS. The flavor of sweet cherry juice changed after LAB fermentation and the fruity odor decreased overall. *Lactobacillus acidophilus* and *Lactobacillus plantarum* significantly increased 2-heptanone, ethyl acetate, and acetone contents, bringing about a creamy and rummy-like favor, whereas *Lactobacillus rhamnosus* GG significantly increased 2-heptanone, 3-hydroxybutan-2-one, and 2-pentanone contents, generating cheesy and buttery-like odors. Principal component analysis of GC–IMS data and linear discriminant analysis of E-nose results could effectively differentiate non-fermented sweet cherry juice and the sweet cherry juice separately inoculated with different LAB strains. Furthermore, there was a high correlation between the E-nose and GC–IMS results, providing a theoretical basis to identify different sweet cherry juice formulations and appropriate starter culture selection for fermentation. This study enables more extensive utilization of sweet cherry in the food industry and helps to improve the flavor of sweet cherry products.

## 1. Introduction

Sweet cherries (*Prunus avium* L.) are temperate fruits native to Europe that grow wild worldwide (Blando and Oomah, 2019). The fruits of sweet cherry plants are rich in anthocyanins, phenolic acids, and flavonoids, which have potential health benefits and can ameliorate several diseases such as cancers, cardiovascular disease, and diabetes mellitus. Recently, sweet cherries have become a very popular consumer item in China owing to their excellent taste and sensory properties (Zhang et al., 2019). According to a survey from the United Nations Food and Agriculture Organization (FAO), the yield of sweet cherries in China has experienced an explosive increase, reaching over 400,000 tons in 2017 with the output expected to reach approximately 100 million by 2025 (Zhang et al., 2020). Despite explosion of the sweet cherries industry in China, several factors limit its further development. Namely, sweet cherries are seasonal fruits, and their fragile peel makes them susceptible to spoilage by microorganisms (Chiabrando et al., 2019). Moreover, the traditional form of fresh sweet cherries is not conducive to long-term storage and transportation, thus limiting future development. Hence, it is necessary to develop new food processing technologies to enrich the product categories of sweet cherries.

Lactic acid bacteria (LAB)-fermented foods have been attracting increased attention in recent years owing to their favorable properties (Pontonio et al., 2019; Şanlier et al., 2019). In particular, LAB-fermented foods have been shown to promote human health in various aspects such as immune system activation, cholesterol reduction, and promotion of the digestive system (Mathur et al., 2020). LAB strains have long been utilized in the fermentation of dairy products. However, developing innovative fermented plant-based products has become a particularly important goal with the rising demand for non-dairy foods, particularly among vegans and individuals with high cholesterol (Gustaw et al., 2021). For example, Dimitrellou et al. (2021) developed emmer-based beverages with fruit juices (blueberry, aronia, and grape) that were fermented with *Lactiplantibacillus plantarum*. The emmer-based drinks enriched with fruit juices exhibited significant potential as probiotic carriers offering a potential dairy-free alternative. Fruits contain various components with health benefits (e.g., vitamins, antioxidants, and polyphenols) and offer several advantages for the growth of LAB (Kandylis et al., 2016). LAB-fermented fruits not only increase the nutritional content of the fruit but also add additional flavors to the original basis (Wang et al., 2022). Szutowska (2020) demonstrated an improvement in the phenolic content of LAB-fermented pomegranate juice. In addition, LAB fermentation of apple juice with different strains resulted in the formation of new aroma compounds such as ketones and acetaldehydes, which are essential components of the final flavor of fermented juice products (Chen et al., 2019; Szutowska, 2020). Cherry juice fermented by dairy- and plant-derived LAB exhibited an elevated level of dihydrocaffeic acid, which is a compound with putative biological activity, and an increase of total volatile compounds (Ricci et al., 2019). Liu et al. (2022b) investigated the effects of LAB on the sensory characteristics of goji berry juice, and the juices fermented with *Lactobacillus plantarum* or *Lactobacillus acidophilus* were described as having a “honey” odor and a “sour” taste, whereas the *Lactobacillus helveticus*-fermented goji berry juice presented a “floral” aroma and “sweetness.”

Flavor represents the sensory impression of a fermented fruit juice and is the dominant factor determining the product quality and consumers’ purchasing decisions (Barrett et al., 2010). The flavor substances, including non-volatile and volatile compounds, contribute to the flavor of sweet cherry juice (Ricci et al., 2019). Non-volatile compounds such as organic acids contribute to the taste, while volatile compounds confer the aroma. Gas chromatography–mass spectrometry (GC–MS) is a standard method for detecting and quantifying volatile compounds in the food industry (Fang et al., 2020). Many studies have demonstrated the application of GC–MS in the analysis of plant-based fermentation products. For example, Kokkinomagoulos et al. (2020) used GC–MS to evaluate the aroma compounds in pomegranate alcoholic beverages fermented by various yeast strains. A total of 30 different volatile compounds were detected in the pomegranate alcoholic beverage, including 15 esters, 4 organic acids, 8 alcohols, and 3 terpenes. They further explored the effects of fermentation temperature and the type of sugar added to the pomegranate alcoholic beverage. The major volatile compounds in the pomegranate alcoholic beverage, such as 3-methyl-1-butanol and 2-methyl-1-butanol, were detected by GC–MS (Kokkinomagoulos et al., 2021). Bontsidis et al. (2021) fermented chokeberry juice by *Lactobacillus paracasei* and analyzed the change in volatile compounds using GC–MS. The fermented chokeberry juice was characterized by aromatically desirable volatile compounds such as alcohols and esters. Ricci et al. (2019) also utilized the GC–MS technique to analyze the volatile compounds in LAB-fermented cherry juice. More recently, the GC–ion mobility spectrometry (GC–IMS) technique emerged and has been applied to characterize the aroma of a food product, offering the advantage of distinguishing the differences in the volatile compounds between products (Wen et al., 2022). GC–IMS is an analytical technique that uses the difference in the migration rate of gas-phase ions in an electric field to characterize chemical substances (Wang et al., 2020). GC–IMS combines the excellent separation capacity of GC with the high sensitivity and fast response of IMS, thereby improving the accuracy of qualitative analysis. Gao et al. (2022) monitored the *Lactobacillus rhamnosus* GG-fermented sour cherry juice by headspace (HS)-GC–IMS and found a huge change in the levels of most volatile compounds during the fermentation. Additionally, an electronic nose (E-nose) is a device designed to mimic the human olfactory system (Wang et al., 2019), which has been successfully applied to classify food types by monitoring the aroma profile (Xu et al., 2020). The main advantages of E-nose are that measurements are rapid, convenient, reliable, and accurate, and this device has been validated for distinguishing food flavors. However, the key limitation is that E-nose only assigns volatile compounds to certain categories and cannot identify the specific volatile compounds or obtain quantitative data to clarify differences in compounds among samples (Harper, 2001). We hypothesized that the combination of E-nose and GC–IMS would provide a more comprehensive and accurate technology to distinguish the aroma profile in fermented sweet cherry juice inoculated with different LAB than either technique alone. However, few studies have distinguished and/or evaluated fermented sweet cherry juice by the integrated analysis of E-nose and GC–IMS.

To evaluate this combined approach in the development of a fermented sweet cherry juice, in this study, we selected three representative LAB species, *Lactobacillus acidophilus*, *Lactobacillus plantarum*, and *Lactobacillus rhamnosus* GG, to ferment sweet cherry juice and explore the changes in terms of physicochemical characteristics and aroma properties. Recently, many studies have reported the outstanding fermentation characteristics of these strains in various fermented fruit beverages. For example, Li et al. (2021a) showed that jujube juices fermented with *Lactobacillus acidophilus* and *Lactobacillus plantarum* served as excellent matrices for the growth of these LAB species, which dramatically increased the total phenolic content and antioxidant activities in the juices. Furthermore, various volatile compounds were identified and increased in total content during fermentation by these LAB in the jujube juices. Moreover, Gao et al. (2022) showed that sour cherry juice fermented by *Lactobacillus rhamnosus* GG had a markedly increased ketones content, which enriched the fruit flavor of the fermented sour cherry juice. Chen et al. (2019) evaluated the influence of these three LAB strains on the flavor profile of fermented apple juice. All three strains exhibited good capacities for growth in the apple juice, and some new compounds, including acetaldehydes and ketones, were generated from the fermentation. In this study, we evaluated the aroma properties of sweet cherry juice separately fermented by different LAB starters using both E-nose and GC–IMS. Additionally, multivariate analysis techniques such as principal component analysis (PCA) and linear discriminant analysis (LDA) were applied to mine valuable information from the E-nose and GC–IMS data, respectively. Finally, the correlation between the E-nose and GC–IMS results was assessed by Spearman’s correlation analysis. Our findings will facilitate the selection of optimal starter cultures to promote flavor development in sweet cherry juice and provide a theoretical basis for this integrated analytical approach to rapidly detect product flavor differences.

## 2. Materials and methods

### 2.1. Chemicals and regents

Analytical standards of citric acid, succinic acid, malic acid, ascorbic acid, and lactic acid were purchased from Aladdin Technology Co., Ltd. (Shanghai, China). Shikimic acid and fumaric acid were obtained from Solabiao Technology Co., Ltd. (Beijing, China). Other reagents such as De Man, Rogosa, Sharpe (MRS) medium; 3,5-dinitrosalicylic acid; and Na_2_CO_3_ were obtained from Sinopharm Chemical Reagent Co. Ltd. (Shanghai, China).

### 2.2. Bacteria reactivation

*Lactobacillus acidophilus* JYLA-16, *Lactobacillus plantarum* JYLP-375, and *Lactobacillus rhamnosus* GG JYLR-005 were obtained in lyophilized form from Zhongke Jia-yi Bioengineering Co., Ltd. (Shandong, China). These strains were stored as a freeze-dried powder at −80°C. The culture was incubated at 37°C for 16 h with 100 ml MRS medium for activation. Subsequently, the activated cells were centrifuged and washed with sterile water to prepare for fermentation.

### 2.3. Sweet cherry juice fermentation

Fully ripened and frozen sweet cherries were obtained from a cherry farm in Shandong, China. The sweet cherries were cleaned and the pulps were removed with a pitter. The obtained pulps were squeezed by a juicer (Midea, China), filtered through four-layer gauze, and centrifuged to obtain clear sweet cherry juice. The obtained juice was pasteurized (72°C, 15 min) and stored at −80°C before fermentation. An initial concentration of 1 × 10^7^ LAB cells/ml was allotted into the pasteurized sweet cherry juice and incubated under a static condition at 37°C for 48 h. All fermentation and analyses were employed in triplicate. The experimental samples were divided into the following four groups for comparison: sweet cherry juice without LAB (CJ), sweet cherry juice fermented with *Lactobacillus acidophilus* JYLA-16 (LAFCJ), sweet cherry juice fermented with *Lactobacillus plantarum* JYLP-375 (LPFCJ), and sweet cherry juice fermented with *Lactobacillus rhamnosus* GG JYLR-005 (LRFCJ).

### 2.4. Physicochemical characteristics and cell viability analyses

#### 2.4.1. Viable cell count

Viable counts of fermented sweet cherry juice were conducted based on the spread-plate method as reported previously (Kaprasob et al., 2017). In brief, serial dilutions of the juice with normal saline were prepared, and aliquots of 0.1 ml of diluted samples were placed on the MRS agar medium and incubated for 36–48 h at 37°C. Plates containing 30–300 colonies were filtered out and recorded as log colony-forming units (CFU)/ml.

#### 2.4.2. Soluble solids content, reducing sugars content, and pH measurements

The SSC of fermented sweet cherry juice was evaluated in units of °Brix using a digital refractometer (3nh, China). The reducing sugar content was determined according to the 3,5-dinitrosalicylic acid (DNS) colorimetric method, as described previously (Kokkinomagoulos et al., 2020). In brief, 2 ml of the diluted juice sample (dilution factor of 500) was mixed with 2 ml DNS solution and heated in boiling water for 5 min. After the mixture cooled down to room temperature, 6 ml of ddH_2_O was added and the absorbance was recorded at 540 nm on an ultraviolet-spectrophotometer (INESA, China); d-glucose was used as a standard. The pH was determined by a digital pH meter (Lei-Ci, China). To prevent contamination of the samples, 50 ml of the sample was removed and placed into a 100 ml flask, and then the pH was detected in the flask.

#### 2.4.3. Ultra-high-performance liquid chromatography (UPLC)-tandem mass spectrometry (MS/MS) analysis of organic acids

To identify the organic acids of fermented sweet cherry juice, a UPLC coupled with linear trap quadrupole–Orbitrap mass spectrometry system (Thermo Fisher Scientific, Waltham, MA, United States) was utilized according to the manufacturer instructions. Briefly, 1 ml of each sample was centrifuged at 12000 rpm for 10 min. The obtained supernatant was filtered through a 0.22 μm membrane for UPLC-MS/MS analysis. The separation of analytes was carried out on a Hypercarb column (100 × 2.1 mm, Thermo Fisher) at 40°C. Linear-gradient elution was performed at a flow rate of 0.25 ml/min. The gradient elution consisted of water (phase A) and acetonitrile (phase B) as mobile phases varying as follows: 0–6 min, 95% B; 6–6.1 min, 55% B; 6.1–10 min, 95% B. An electrospray ion source was used to scan in multiple reaction monitoring positive- and negative-ion modes. The MS conditions were as follows: source temperature, 130°C; desolvation temperature, 500°C; flow rate of the desolvation gas, 800 l/h; flow rate of the cone gas (N_2_), 150 l/h; capillary voltage, 3 kV; and collision gas, argon (99.99%). Compounds were identified by their retention times and ion pair strength. The samples were quantified based on their peak areas using calibration curves for the different standard concentrations.

### 2.5. E-nose analysis

The aroma characteristics were analyzed using an E-nose system as previously described (Ni et al., 2021) with some modifications. E-nose analysis was conducted on a SuperNose system (Isenso Group Cooperation, United States) equipped with 14 metal oxide gas sensors. The gas sensors system demonstrated good selectivity of different types of volatile organic compounds (Table 1). The sweet cherry juice (5 ml) was incubated at 45°C for 30 min to evaporate the odorants prior to E-nose analysis. The sampling was conducted at 0.6 l/min for 60 s. A clean stage was used for each sample by pumping clean air at 1.0 l/min to blank sensor signals. Six replicate measures were taken for each sample and the last three records were adopted for further analysis.

**Table 1:** General description of electronic nose sensor array.

Sensor No.	Sensitive property of the E-nose sensors
S1	Ammonia and amines
S2	Hydrogen sulfide and sulfides
S3	Hydrogen
S4	Organic solvents and ethanol
S5	Ketones, alcohols, aldehydes, and aromatic compounds
S6	Methane, biogas, and natural gas
S7	Flammable gases
S8	Volatile organic compounds
S9	Liquid gas and natural gas
S10	Liquid gas and flammable gases
S11	Alkanes, ethanol, natural gas, and smoke
S12	Ethanol and organic solvents
S13	Smoke and cooking odor
S14	Methane and natural gas

The description for the sensor array was from the handbook of the E-nose (Isenso Group Cooperation, United States).

### 2.6. Headspace gas chromatography–ion mobility spectrometry analysis

The HS-GC–IMS analysis was conducted on the FlavourSpec^®^ GC-IMS device (G.A.S. Dortmund, Germany). Sample analysis was performed according to a previously reported method (Chen et al., 2020a) with some modifications. First, 2 ml of each sample was deposited into a headspace bottle and heated at 60°C for 15 min for volatilization. Subsequently, 500 μl of the sample headspace was pumped into a heated injector by nitrogen (99.99% purity) at 85°C. The volatile compounds were first separated through the MXT-5 capillary column (15 m × 0.53 mm). The carrier gas was supplied at an initial flow rate of 2 ml/min for 2 min, which was subsequently increased to 100 ml/min for 18 min. The volatile compounds were then driven into an ionization chamber and the second separation step proceeded by ionization in the IMS ionization chamber. The retention index (RI) of volatile compounds was calculated using the n-ketones of C4–C9 as external references. The volatile compounds were identified by comparing the RI and drift time with those in the GC-IMS NIST 11 library. Moreover, the compounds were quantified according to the peak intensity.

### 2.7. Statistical analysis

The results are presented as mean ± standard deviation of three independent experiments. One-way analysis of variance and Duncan’s test were applied in SPSS 18 (SPSS Inc., United States), and *p* < 0.05 was regarded as statistically significant. LDA was performed using the response values of E-nose sensors and data were plotted using ISENO software. PCA was conducted using the peak signal intensities detected by GC–IMS and plotted with SIMCA software (Version 14.1, Umetrics, Sweden). To assess the correlation between E-nose sensor responses and volatile compound levels detected by GC–IMS, Spearman’s correlation analysis was performed with Origin Pro 2021 software (Origin Lab Corporation, Northampton, MA, United States).

## 3. Results and discussion

### 3.1. Physicochemical properties of sweet cherry juice during fermentation

**Figure 1:**
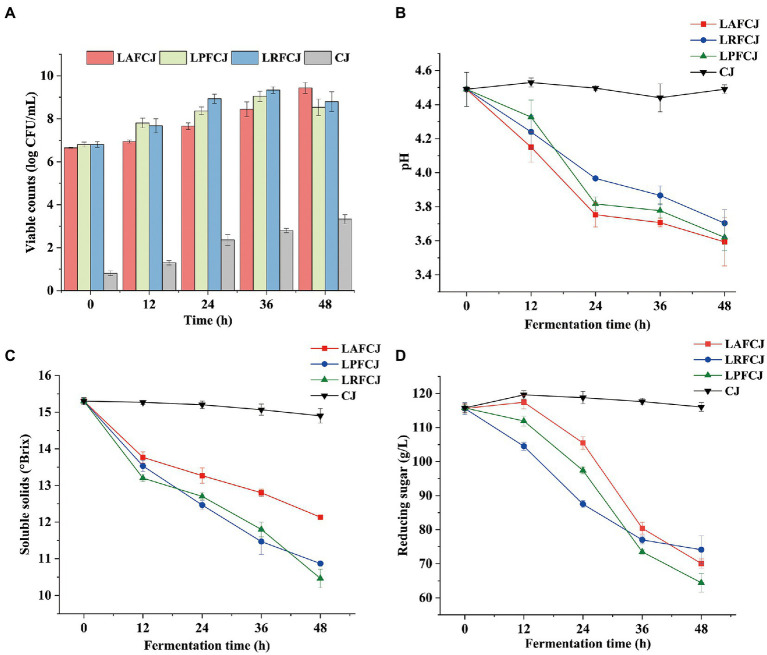
Physicochemical properties change in non-incubated sweet cherry juice and sweet cherry juice fermented with different lactic acid bacteria (LAB) strains. **(A)** The population change of LAB. Note: The viable LAB can also be detected in the CJ. **(B)**The pH value. **(C)** The total soluble solids content (SSC) content. **(D)** The reducing sugar contents.

The count of viable bacteria in sweet cherry juice represents a good indicator of the quality of fermented products, because the number of viable cells is directly related to the success of fermentation (Shi et al., 2022). The changes in viable counts during fermentation are depicted in Figure 1A. All three of the selected LAB strains showed no significant lag phase throughout the fermentation process, indicating that sweet cherry juice is an appropriate substrate for LAB. During the first 24 h of fermentation, the viable counts of all three LAB strains rapidly increased. However, there was a noticeable drop in the microbial population of the LPFCJ and LRFCJ groups after 36 h. This may have been be due to the low pH environment generated during fermentation, which inhibits bacterial growth (Ruiz Rodríguez et al., 2019). The LAFCJ sample showed relatively less variation in viable counts compared to the other two groups, which is likely due to the strong acid tolerance of *Lactobacillus acidophilus* (Chen et al., 2019). In general, after 48 h fermentation, the number of viable bacteria of all fermented juice samples reached over 8.5 log CFU/ml, which is significantly higher than the baseline count (8.0 log CFU/ml) for probiotic products recommended by the FAO/WHO (2002).

Changes in pH during fermentation are shown in Figure 1B. There was a slight decrease in pH observed after 12 h, irrespective of the LAB strain used in fermentation. Moreover, the pH of LAFCJ and LPFCJ samples decreased more rapidly during the first 24 h of fermentation, followed by a more gradual decrease until 48 h. A decrease in pH is generally associated with the production of organic acids by LAB during their energy metabolism and growth processes (Lee et al., 2019). After 48 h of fermentation, LAFCJ showed more acid-producing activity than LPFCJ and LRFCJ. The more robust acidity of sweet cherry juice fermented with *Lactobacillus acidophilus* is potentially due to its high efficiency in metabolizing carbohydrates into lactic acids. These findings are in line with the results reported for cashew juice fermentation (Kaprasob et al., 2017).

Sugar content variation in the sweet cherry juice can be measured with respect to the SSC or reducing sugar content. Figures 1C,D depict the changes in SSC and reducing sugar contents of fermented sweet cherry juice. In all fermented juices, the levels of SSC and reducing sugars decreased throughout fermentation. Moreover, the decreases of SSC and reducing sugars in LPFCJ were more significant than those in LAFCJ and LRFCJ. These results agree with the findings of Degrain et al. (2020), who reported that the decrease in total soluble solids in fermented African nightshade was more significant when using *Lactobacillus plantarum* than with other LAB strains. The extent of sugar reduction was considerable owing to the utilization of sugar to support bacterial growth and for the metabolism into lactic acid.

### 3.2. Characterization of the organic acids profile

Characterization of the organic acid profile in fermented foods is crucial for determining the sensory properties and serves as an indicator of the bacterial activity of the cultures and probiotics. The levels of organic acids generated in the sweet cherry juice during fermentation are shown in Table 2. Seven organic acids were detected, including lactic acid, malic acid, citric acid, l-ascorbic acid, shikimic acid, fumaric acid, and succinic acid.

**Table 2:** The organic acids profiles of lactic acid bacterial fermented sweet cherry juice.

Organic acid (mg/L)	CJ	LAFCJ	LPFCJ	LRFCJ
Fumaric acid	139.33 ± 29.01^a^	ND	ND	ND
Succinic acid	87.70 ± 6.81^a^	48.77 ± 6.61^b^	38.96 ± 4.13^c^	2.25 ± 0.93^d^
Malic acid	323.15 ± 6.16^a^	ND	ND	0.54 ± 0.19^b^
Shikimic acid	424.24 ± 186.71^b^	572.88 ± 9.74^ab^	702.51 ± 33.27^a^	534.95 ± 13.88^ab^
L-ascorbic acid	3077.50 ± 1651.66^b^	5686.73 ± 00.82^a^	6110.48 ± 602.40^a^	4453.75 ± 261.14^ab^
Citric acid	57.91 ± 25.72^a^	30.00 ± 0.97^b^	35.62 ± 2.72^ab^	58.96 ± 2.69^a^
Lactic acid	ND	8225.13 ± 173.41^a^	6809.85 ± 507.09^b^	4295.89 ± 56.62^c^

Results are the mean value of three replicates. Values in the same column with different superscript letters are significantly different (*p* < 0.05). ND means the concentration of given compound was below the limit of detection.

Lactic acid was the most typical organic acid generated during fermentation, and its concentration increased sharply after fermentation. Among all fermented samples, LAFCJ had the highest lactic acid content (8225.13 ± 173.41 mg/l), whereas LRFCJ had the lowest lactic acid content (4295.89 ± 56.62 mg/l). Numerous studies have demonstrated lactic acid as the primary fermentation product, including in the LAB fermentation of elderberry, black chokeberry, and sea buckthorn juices (Markkinen et al., 2019; Cirlini et al., 2020). The lactic acid concentration depends on the raw material matrix and the specific LAB strains applied. The decomposition of sugars in the fermentation matrix is the leading cause of lactic acid production. Notably, lactic acid and other organic acids produced by LAB can function as bio-preservatives with additional components such as carbon dioxide and bacteriocins. This is likely because the acidic environment they create can destroy the cell membrane of spoilage germs (Mendes Ferreira and Mendes-Faia, 2020). Hence, the plentiful lactic acid produced by LAB could favor the preservation of sweet cherry juice.

Malic acid is one of the primary organic acids in sweet cherry that provides the characteristic sourness and acts as a flavor blender to create a strong and sharp taste (Peng et al., 2021). The CJ samples produced 323.15 ± 6.16 mg/l malic acid. However, all fermented samples showed a dramatic decrease in malic acid content, with LAFCJ and LPFCJ exhibiting the most marked decline. The downward trend of malic acid closely paralleled the generation of lactic acid, which demonstrated that malolactic metabolism occurred in all fermented samples. Moreover, the malolactic transition was more pronounced in LAFCJ, consistent with a previous observation in fermented apple juice (Chen et al., 2019).

The trend of citric acid content varied depending on the LAB species utilized for fermentation. LAFCJ (30.00 ± 0.97 mg/l) and LPFCJ (35.62 ± 2.7 mg/l) samples showed lower citric acid levels than the unfermented (CJ) samples (57.91 ± 25.72 mg/l). This is possibly because *Lactobacillus acidophilus* and *Lactobacillus plantarum* utilize citric acid and malic acid as carbon sources. Previous studies have shown that LAB can use citric acid and malic acid simultaneously, even though their relative conversion rates differ. In general, citric acid metabolism is accompanied by the use of carbohydrates or other energy sources. LAB can convert citric acid into acetate and oxaloacetate by ATP-citrate lyase (Cirlini et al., 2020). The results of this study suggest that *Lactobacillus acidophilus* may be more prone to use citric acid than the other two LAB strains, and the citric acid content in LAFCJ samples was consistently the lowest. Conversely, the citric acid content of LRFCJ samples showed an increase after fermentation, which has also been reported with fermentation of carrot juice (Landete et al., 2013). The increase of the citric acid content in the LRFCJ samples may be ascribed to the hydrolytic activity of the microorganism, which can induce the higher release of citric acid from sweet cherry juice (Delgado-Ospina et al., 2020).

l-Ascorbic acid (vitamin C) is one of the primary hydrosoluble antioxidants of the fruit and is an essential component of human nutrition (Zheng et al., 2022). A previous study revealed that LAB fermentation could protect l-ascorbic acid from oxidation, thereby retaining the freshness of fruits and vegetables (Bergqvist et al., 2005). Our results demonstrated that after fermentation by LAB, the l-ascorbic acid content increased sharply, and the LPFCJ samples exhibited the highest l-ascorbic acid content (6110.48 ± 602.40 mg/l), which was almost double that of the control group. The increase in l-ascorbic acid in the fermented sweet cherry juice might be attributed to the fact that ascorbic acid can be produced by these LABs (Kaprasob et al., 2017). Shikimic acid is a metabolite found in plants that serves as a biosynthetic intermediary for the aromatic amino acids (phenylalanine, tyrosine, and tryptophan), which are vital components of human nutrition (Wu et al., 2022). The shikimic acid content in sweet cherry juice also increased after fermentation, which is possibly due to the bioconversion of phosphoenolpyruvate to shikimic acid in the shikimate pathway (Li et al., 2021b). All of the fermented sweet cherry juice samples displayed a significant decline in fumaric acid and succinic acid contents, which may be related to microbial metabolism, especially the tricarboxylic acid cycle (Yamamoto et al., 2021).

### 3.3. Aroma profile from E-nose measurements

**Figure 2:**
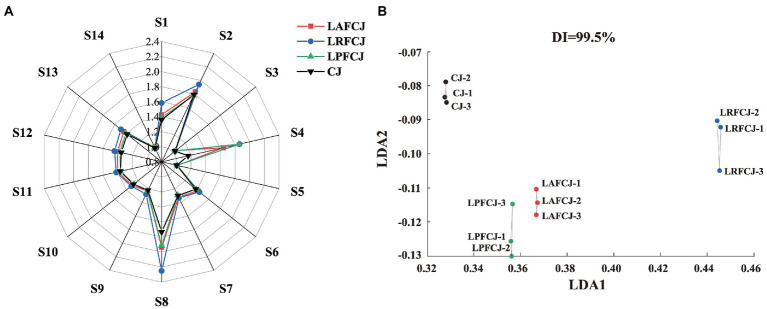
The E-nose analysis of volatile profile in non-incubated sweet cherry juice and sweet cherry juice fermented with different LAB strains. **(A)** The sensors responding radar data from E-nose. **(B)** The linear discriminant analysis (LDA) analysis from E-nose.

E-nose analysis was conducted to distinguish the aroma profiles of juices incubated with different LAB strains. Figure 2A shows the responses of the 14 sensors to the juice samples. High responses were observed in the sensors S1, S2, S4, and S8, with the volatile organic compound sensor (S8) showing the most extensive response value, which identified the specific aroma substances of sweet cherries (aldehydes, alcohols, and esters).

Linear discriminant analysis (LDA) is a classical supervised data dimension reduction method, which can be utilized to identify perceptible olfactory differences between different sources, narrowing the differences between similar sources and widening the differences between distinct sources, with more considerable distances between groups indicating more significant variability (Wang et al., 2021a). As shown in Figure 2B, LDA clustered different samples with good differentiation according to the fermentation strain: CJ is concentrated at the top of the figure and LAFCJ and LPFCJ are at the bottom. Additionally, the reproducibility between the three parallel measurements in each group was good, with no significant differences observed. According to the LDA results, there were significant differences in the aroma intensity of the cherry juices fermented with different LAB, which indicated that the aroma profiles between different samples can be effectively identified with the E-nose.

### 3.4. Headspace gas chromatography–ion mobility spectrometry analysis of volatile compounds

#### 3.4.1. Headspace gas chromatography–ion mobility spectrometry topographic plots

**Figure 3:**
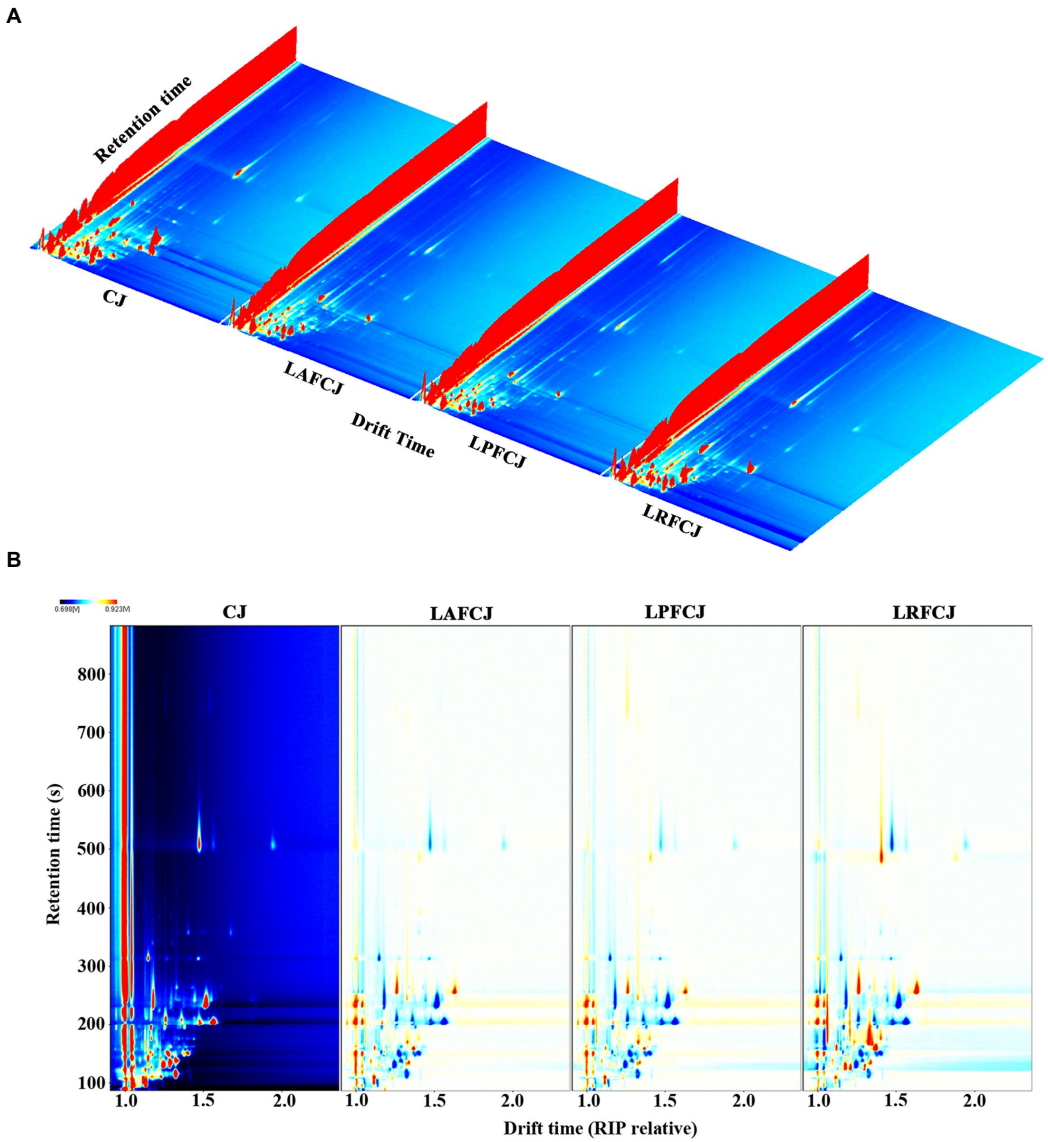
The headspace gas chromatography–ion mobility spectrometry (HS-GC-IMS) topographic plots of volatile profile in in non-incubated sweet cherry juice and sweet cherry juice fermented with different LAB strains. **(A)** The 3D topography of volatiles. **(B)** The 2D topography of volatiles.

The differences in volatile compounds in sweet cherry juice fermented by different LAB were further analyzed by HS-GC–IMS. Figure 3A exhibits a three-dimensional topographical visualization of the identified volatile compounds, demonstrating comparable compounds identified in the different samples. Nevertheless, the signal intensities presented a wide range of differences. To further analyze and evaluate the differences between samples, a two-dimensional top-view plot was constructed (Figure 3B). The entire spectrum covered the total headspace substances of the samples, with the majority of signals appearing at the retention time of 100–900 s and drift time of 1.0–1.8 ms. The signal strength of the material is represented by its color, with red indicating higher intensity and white indicating lower intensity; the greater the intensity, the darker the color. As shown in Figure 3B, the GC–IMS analysis suggested that most compounds exhibited a huge change in these fermented samples, with some increasing and some declining compared to their contents in the unfermented CJ. This variation indicated different metabolic footprints of different LAB in sweet cherry juice under corresponding fermentation conditions.

#### 3.4.2. Qualitative analysis of volatile compounds

**Figure 4:**
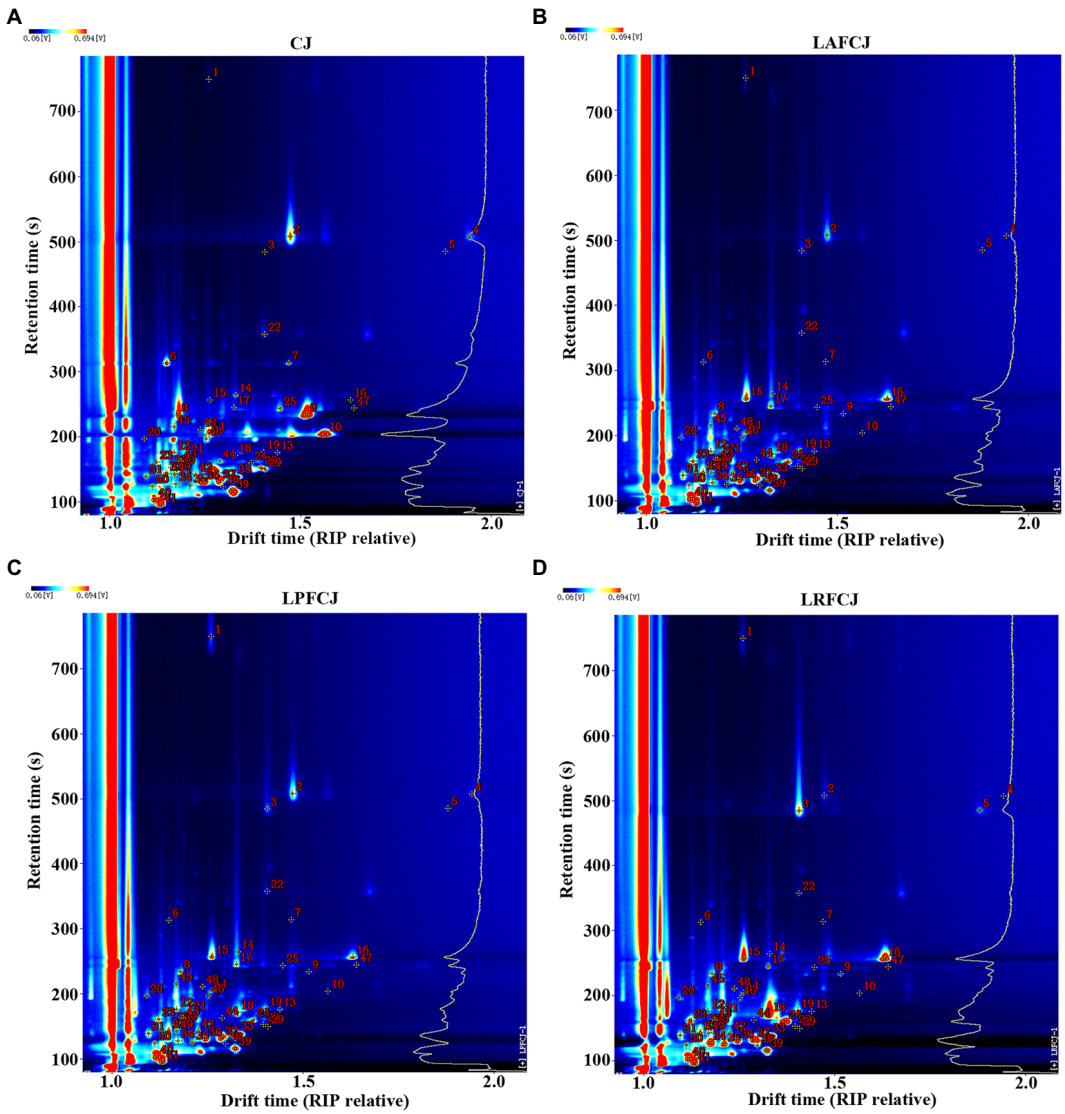
The ion migration spectra of volatiles detected by HS-GC-IMS. **(A)** The ion migration spectra of volatiles in CJ. **(B)** The ion migration spectra of volatiles in LAFCJ. **(C)** The ion migration spectra of volatiles in LPFCJ. **(D)** The ion migration spectra of volatiles in LRFCJ.

The qualitative results of the volatile compounds of fermented sweet cherry juice with the three LAB strains are presented in Figure 4; Table 3. HS-GC–IMS detected 50 signal peaks and 42 typical compounds were identified. However, there were eight compounds that could not be qualitatively identified due to the limited data of the library database. Based on the identified compounds, the 42 distinct volatile compounds included 1 acid, 12 alcohols, 16 aldehydes, 3 esters, and 10 ketones. Since monomer ions and neutral molecules may form auxiliary substances in the drift area, various single compounds may generate multiple signals, resulting in the detection of monomers and dimers for the same substance. According to Chen et al. (2020b), many monomers with proton affinity absorb some reactant protons before combining to create dimers. Furthermore, several volatile compounds such as E-2-hexenol, hexanal, and ethyl acetate could generate different signals. For example, Li et al. (2019) illustrated that the same volatile compounds present at different concentrations can generate diverse signals in *Tricholoma matsutake* Singer. Additionally, a newly formed dimer exhibits a much greater molecular weight than the monomer and produces multiple signals.

**Table 3:** Identification of volatile compounds in non-inoculated sweet cherry juice and sweet cherry juice inoculated with different LAB strains by GC-IMS.

Count	Compound	CAS#	Classification	Formula	MW	RI	Rt [sec]	Dt [a.u.]	Odor description
1	Linalool oxide (pyranoid)	14,049-11-7	Alcohols	C_10_H_18_O_2_	170.3	1276.9	749.103	1.26154	Floral, honey
2	Nonanal-M	124-19-6	Aldehydes	C_9_H_18_O	142.2	1108.7	507.332	1.47509	Citrus, rose, lemon peel
3	2-nonanone-M	821-55-6	Ketones	C_9_H_18_O	142.2	1092.6	484.176	1.40911	Cheesy, green, coconut
4	Nonanal-D	124-19-6	Aldehydes	C_9_H_18_O	142.2	1108.3	506.651	1.94557	Citrus, rose, lemon peel
5	2-nonanone-D	821-55-6	Ketones	C_9_H_18_O	142.2	1093.1	484.857	1.88134	Cheesy, green, coconut
6	Benzaldehyde-M	100-52-7	Aldehydes	C_7_H_6_O	106.1	957.9	313.034	1.15176	Almond, fruity
7	Benzaldehyde-D	100-52-7	Aldehydes	C_7_H_6_O	106.1	958.8	313.725	1.47143	Almond, fruity
8	(E)-2-hexenal-M	6,728-26-3	Aldehydes	C_6_H_10_O	98.1	848	233.253	1.18169	Green, clean, fruity
9	(E)-2-hexenal-D	6,728-26-3	Aldehydes	C_6_H_10_O	98.1	848.7	233.598	1.51812	Green, clean, fruity
10	Hexanal-D	66-25-1	Aldehydes	C_6_H_12_O	100.2	792.6	203.551	1.56721	Green, fruity
11	Hexanal-M	66-25-1	Aldehydes	C_6_H_12_O	100.2	794.5	204.587	1.2631	Green, fruity
12	3-Methyl-3-buten-1-ol-M	763-32-6	Alcohols	C_5_H_10_O	86.1	725.8	175.384	1.17079	Sweet fruity
13	3-Methyl-3-buten-1-ol-D	763-32-6	Alcohols	C_5_H_10_O	86.1	726.9	175.814	1.44178	Sweet fruity
14	Heptanal	111-71-7	Aldehydes	C_7_H_14_O	114.2	901.3	264.71	1.33321	Fresh, green, cognac
15	2-heptanone-M	110-43-0	Ketones	C_7_H_14_O	114.2	890.8	256.182	1.26501	Cheesy, green, creamy
16	2-heptanone-D	110-43-0	Ketones	C_7_H_14_O	114.2	891.9	256.751	1.63278	Cheesy, green, creamy
17	n-Hexanol-M	111-27-3	Alcohols	C_6_H_14_O	102.2	870.1	245.097	1.32786	Fruity, sweet, green
18	3-hydroxybutan-2-one	513-86-0	Ketones	C_4_H_8_O_2_	88.1	715.5	171.191	1.33188	Sweet, buttery, creamy
19	1	Unidentified	Unidentified	*	0	729.5	176.876	1.40543	
20	3-Methyl-2-butenal	107-86-8	Aldehydes	C_5_H_8_O	84.1	779.2	197.041	1.09341	Sweet, fruity, pungent
21	2	Unidentified	Unidentified	*	0	713.2	170.275	1.20717	
22	Octanal	124-13-0	Aldehydes	C_8_H_16_O	128.2	1004.6	357.514	1.40823	Citrus, green, and peely
23	2-Pentanone-M	107-87-9	Ketones	C_5_H_10_O	86.1	690.3	160.965	1.1247	Sweet, fruity, banana
24	2-Pentanone-D	107-87-9	Ketones	C_5_H_10_O	86.1	685.9	159.551	1.37303	Sweet, fruity, banana
25	3	Unidentified	Unidentified	*	0	866.6	243.241	1.44962	
26	2-methylbutanal-M	96-17-3	Aldehydes	C_5_H_10_O	86.1	661.6	153.004	1.16554	Cocoa, coffee, nutty
27	3-methylbutanal-M	590-86-3	Aldehydes	C_5_H_10_O	86.1	645.8	148.736	1.17836	Chocolate, peach, fatty
28	2-methylbutanal-D	96-17-3	Aldehydes	C_5_H_10_O	86.1	661.6	153.004	1.3991	Cocoa, coffee, nutty
29	3-methylbutanal-D	590-86-3	Aldehydes	C_5_H_10_O	86.1	646.5	148.93	1.4099	Chocolate, peach, fatty
30	1-butanol	71-36-3	Alcohols	C_4_H_10_O	74.1	664.1	153.683	1.18241	Balsamic, whiskey
31	Ethyl Acetate-M	141-78-6	Esters	C_4_H_8_O_2_	88.1	608	138.537	1.09827	Sweet, grape, rummy
32	2-butanol	78-92-2	Alcohols	C_4_H_10_O	74.1	624.8	143.089	1.1509	Sweet, apricot
33	Ethyl Acetate-D	141-78-6	Esters	C_4_H_8_O_2_	88.1	610.9	139.341	1.33042	Sweet, grape, rummy
34	2,3-butanedione	431-03-8	Ketones	C_4_H_6_O_2_	86.1	566.6	127.381	1.17613	Buttery, sweet, creamy
35	4	Unidentified	Unidentified	*	0	557.6	124.971	1.2129	
36	2-Butanone	78-93-3	Ketones	C_4_H_8_O	72.1	576.5	130.058	1.24679	Ethereal, fruity
37	5	Unidentified	Unidentified	*	0	583.4	131.933	1.29077	
38	6	Unidentified	Unidentified	*	0	550.4	123.007	1.30014	
39	Tert-butanol	75-65-0	Alcohols	C_4_H_10_O	74.1	522.8	115.574	1.32567	Camphor
40	Acetone	67-64-1	Ketones	C_3_H_6_O	58.1	476	102.94	1.11991	Ethereal, apple, pear
41	Ethanol	64-17-5	Alcohols	C_2_H_6_O	46.1	446.2	94.906	1.13553	Alcoholic
42	7	Unidentified	Unidentified	*	0	623	142.599	1.22711	
43	Pentanal	110-62-3	Aldehydes	C_5_H_10_O	86.1	693.6	162.313	1.18935	Bready, fruity, berry
44	8	Unidentified	Unidentified	*	0	693.3	162.19	1.29091	
45	Butanoic acid	107-92-6	Acids	C_4_H_8_O_2_	88.1	814.5	215.272	1.1705	Cheesy, buttery, fruity
46	Pentan-1-ol	71-41-0	Alcohols	C_5_H_12_O	88.1	779.3	197.076	1.25531	Balsamic, bready
47	n-Hexanol-D	111-27-3	Alcohols	C_6_H_14_O	102.2	867.8	243.83	1.64196	Fruity, sweet, green
48	Butyl acetate	123-86-4	Esters	C_6_H_12_O_2_	116.2	806.5	210.975	1.23909	Fruity, banana
49	2-Methyl-1-propanol	78-83-1	Alcohols	C_4_H_10_O	74.1	622.3	142.408	1.1719	Ethereal, winey
50	1-Propanol	71-23-8	Alcohols	C_3_H_8_O	60.1	551.7	123.361	1.11336	Slightly sweet, fruity

M indicates monomer and D indicates dimer. MW represents the molecular weight of the volatile compounds. RT represents the retention time in the capillary GC column. RI represents the retention indexes of the volatile compounds in the GC column. Dt represents the drift time in the drift tube. The ordinal numbers in Table 3 corresponded to those in Figure 4. Odor descriptions were cited from http://www.thegoodscentscompany.com/index.html.

#### 3.4.3. Fingerprint analysis of volatile compounds

**Figure 5:**
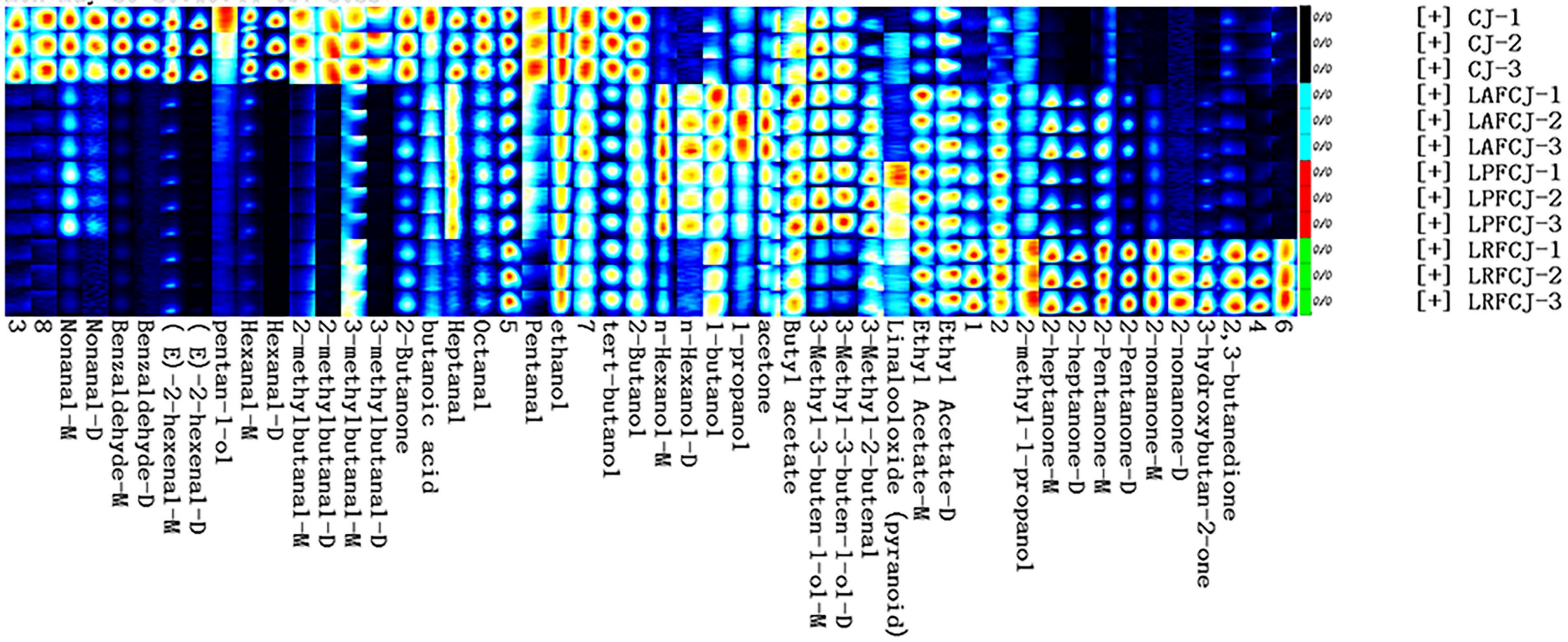
The fingerprint plot of volatiles. M indicates monomer and D stands for dimer. The fingerprint encompasses all detectable signals, where each row represents a sample, and each column represents a substance. Unknown chemicals were labeled with numbers, whereas known substances were labeled with their existing names.

For a more intuitive presentation of the distinction of volatile compounds among samples, fingerprint analysis was conducted (Figure 5). The results indicated that alcohols, aldehydes, esters, and ketones were the primary groups of volatile flavor compounds produced throughout fermentation of sweet cherry juice (Figure 5; Supplementary Table S1). The aldehydes content has a direct impact on flavor, thus affecting the consumer acceptance of foods. A higher aldehydes content may cause an unpleasant flavor in food (Liu et al., 2022a), whereas a lower content can bring about a pleasant flavor. Melgarejo et al. (2011) analyzed the volatile compounds in pomegranates from nine Spanish cultivars, showing that the BA1 cultivar with a high level of aldehydes, accounting for over 50% of the total volatiles, had poor overall consumer ratings. However, the ME1 cultivar with a low level of aldehydes, accounting for under 40% of the total volatiles, had higher overall preference ratings in the consumer panel. In the present study, the major aroma compounds of the CJ samples were (E)-2-hexenal and hexanal, which confer a green and fruity aroma, including the characteristic aroma of sweet cherry (Nunes et al., 2022). However, in the fermented sweet cherry juices, the levels of the majority of aldehydes decreased compared to those of the CJ, which was consistent with previous fermentation investigations in fermented sour cherry juice, watermelon juice, and pomegranate juice (Dicagno et al., 2017; Mandha et al., 2021; Gao et al., 2022).

The entire fermentation process can neutralize undesirable flavors and improve product acceptance. Alcohols play a crucial role in flavor formation since they not only have a pleasant flavor but can also dissolve other aroma compounds. In this study, the contents of most alcohols did not substantially change before and after fermentation. However, a previous study showed that the contents of several alcohols increased during fermentation of sour cherry juice by *Lactobacillus rhamnosus* GG (Gao et al., 2022). Esters, which are closely associated with the fruity and sweet flavor, also appear to have played an essential role in the fermentation of sweet cherry juice. Previous studies showed that LAB turned the carbohydrates into their end products during fermentation, including organic acids and alcohols (Wang et al., 2021b). Several esters, including ethyl acetate, could be formed by the combination of organic acids and alcohols. Cai et al. (2019) revealed that throughout the liquor brewing process, more ethyl acetate was produced during fermentation and became a crucial volatile component. Ethyl acetate was also identified as one of the most abundant esters in pomegranate alcoholic beverages, which was detected in all fermented samples (Kokkinomagoulos et al., 2020, 2021). In the present study, two esters (ethyl acetate and butyl acetate) were identified, which exhibited a slight increase after fermentation. However, Gao et al. (2022) detected three types of esters in *Lactobacillus rhamnosus* GG-fermented sour cherry juice, including ethyl acetate, n-propyl acetate, and methyl 2-methylbutanoate, by GC–IMS, and the ethyl acetate content did not change substantially during fermentation, whereas n-propyl acetate generation was enhanced. Bontsidis et al. (2021) reported that the ethyl acetate content of *Lactobacillus paracasei-*fermented chokeberry juice decreased after fermentation.

In the present study, the ketone contents of the CJ samples were low. However, after fermentation, the total ketone content increased sharply and was attributed to the prominent aroma in all fermented juices. Specifically, the contents of ketones, including 2-nonanone, 2-heptanone, 3-hydroxybutan-2-one, and 2-pentanone, increased in all fermented samples in this study, which may be due to the acidic environment caused by LAB, inducing an inhibitory effect on enzymes correlated with β-oxidation (Gao et al., 2022). This phenomenon is also in accordance with the results reported for dairy- and plant-derived LAB-fermented sweet cherry juice (Ricci et al., 2019). Most ketones have a cheesy and butter-like flavor, which can enrich the fruit flavor of fermented sweet cherry juice. Acids are the primary product of LAB fermentation, imparting a new flavor to fermented products while preventing spoilage. The main acid detected by GC–IMS was butanoic acid with a cheesy and buttery flavor, which increased in the LAPCJ and LPFCJ samples but decreased in the LRFCJ samples. Moreover, the previous study in *Lactobacillus rhamnosus* GG-fermented sour cherry juice showed that the primary acid detected by GC–IMS was 2-methyl-propanoic acid, which decreased gradually from 0 to 12 h but the content fluctuated after 12 h of fermentation (Gao et al., 2022). Conversely, in the dairy- and plant-derived LAB-fermented sweet cherry juice, GC–MS detected acetic acid as the primary acid produced during fermentation (Ricci et al., 2019).

We also detected differences in the aroma of the sweet cherry juices fermented by the three different LAB strains, which may reflect the distinct protein and carbohydrate metabolic pathways of these three strains (Chen et al., 2019). In the LAFCJ samples, the major compounds were 2-heptanone (3591.86 ± 622.68), acetone (2179.23 ± 136.01), ethyl acetate (1995.56 ± 67.74), and 2-pentanone (1577.39 ± 210.05; Supplementary Table S1). The main flavor substances of LPFCJ were similar to those of LAFCJ, with some differences. In the LPFCJ samples, the major compounds were 2-heptanone (2113.88 ± 119.55), ethyl acetate (2064.71 ± 63.68), acetone (1999.45 ± 63.50), and tert-butanol (999.71 ± 55.42; Supplementary Table S1). The high contents of 2-heptanone and ethyl acetate bring about a cheesy, creamy, and rummy-like aroma (Clarke et al., 2019), which significantly contributes to the juice flavor. Acetone is responsible for apple, pear, and ethereal-like aromas, and contributed to the fruity and rummy-like aroma in the LAFCJ and LPFCJ samples. The compound 2-pentanone is characterized by a sweet, fruity, and banana flavor, and its content was enhanced in the LAPCJ samples, thereby increasing the fruit flavor of the fermented sweet cherry juice. Tert-butanol, assigned to camphor, was a characteristic volatile compound in LPFCJ. The major aroma of LRFCJ was derived from ketones such as 2-heptanone (5218.16 ± 42.14), 3-hydroxybutan-2-one (4116.80 ± 346.38), and 2-pentanone (4063.11 ± 41.77), resulting in stronger cheesy, creamy, and buttery-like odors than found in the LAFCJ and LPFCJ samples (Supplementary Table S1).

#### 3.4.4. Principal component analysis

**Figure 6:**
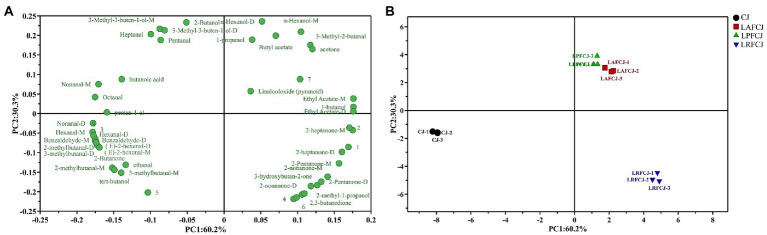
The principal component analysis (PCA) analysis of volatile compounds in non-incubated sweet cherry juice and sweet cherry juice fermented with different LAB strains. **(A)** The loading plot of the identified volatiles. **(B)** The score plot of the identified volatiles.

Principal component analysis (PCA) was performed to highlight the variations in volatile compounds among different LAB-fermented sweet cherry juices. The most significant variable was the first principal component (PC1), which accounted for 60.2% of the total variance. PC1 negatively correlated with most aldehydes (Figure 6A). The unfermented sweet cherry juice (CJ) was positioned on the negative side of the PC1 axis (Figure 6B), indicating a positive correlation between the production of most aldehydes and the unfermented sweet cherry juice. By contrast, the LAB-fermented sweet cherry juices were situated on the positive side of the PC1 axis, demonstrating that PC1 distinguished between the fermented and unfermented sweet cherry juices. In addition, PC2 differentiated the fermented sweet cherry juice according to the starter cultures. The LRFCJ fermented samples were located on the negative PC2 axis and positively correlated with volatile compounds such as 2-heptanone, 2-pentanone, 2-nonanone, and 3-hydroxybutan-2-one. Notably, the LAFCJ and LPFCJ samples were closely clustered, indicating that the aroma profiles of sweet cherry juice inoculated with these two strains were more comparable. Collectively, these results were consistent with the E-nose data (Figure 2).

### 3.5. Correlation between E-nose and GC–IMS results

**Figure 7:**
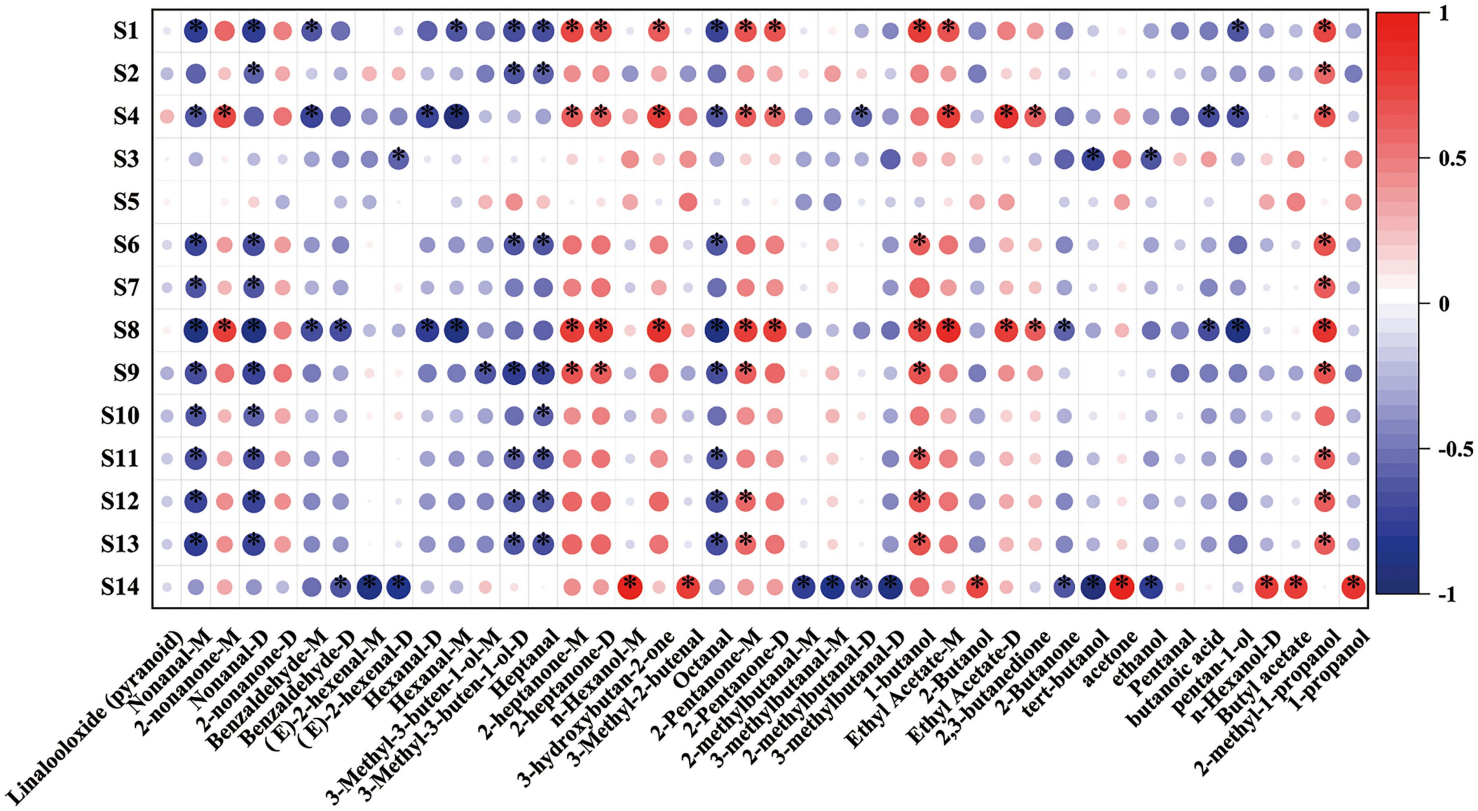
Spearman’s correlation heat map showing the correlation between the volatile compound levels and electronic nose sensor responses. Colors represent correlation coefficients, with red indicating a positive correlation and blue indicating a negative correlation. ^*^Represent significance at *p* < 0.05.

The E-nose analysis and GC–IMS data could both effectively categorize the sweet cherry juices fermented with different LAB strains but to varying degrees. In contrast to the general volatile chemical data provided by E-nose, GC–IMS can detect individual volatile chemicals in samples. Thus, the combination of the two strategies may enhance the overall performance of characterization of fermented juice. Figure 7 shows the correlations between E-nose sensor responses and volatile compound levels detected by GC–IMS. The S4 sensor responded positively to 2-heptanone and ethyl acetate-D, and GC–IMS detected high concentrations of these chemicals in the LPFCJ samples. The S4 sensor also demonstrated significant response intensities to the LPFCJ samples, confirming the E-nose categorization. The S8 sensor was positively correlated with 3-hydroxybutan-2-one, a typical volatile compound identified in the LRFCJ samples. These results demonstrated that the E-nose sensor responses and volatile compound levels identified by GC–IMS could discriminate the distinctive aroma of sweet cherry juices inoculated with different LABs. Furthermore, the substantial association between the E-nose sensor responses and some volatile compound levels could help to hasten the development of an E-nose-based approach for targeted analysis.

## 4. Conclusion

This study shows that sweet cherry is an outstanding fruit substrate for fermentation-based biotransformation into higher-value volatile compounds using the three selected LAB strains (*Lactobacillus acidophilus*, *Lactobacillus plantarum*, and *Lactobacillus rhamnosus* GG). This work indicated that all three strains could grow well in the sweet cherry juice, although their fermentation resulted in different flavor and aroma features. Higher acid production and rapid growth in the juice were found in LAFCJ and LPFCJ samples compared with those of LRFCJ samples. This study thus established the volatile compound fingerprint of sweet cherry fermented by these three LAB strains. Furthermore, the characteristic volatile compounds in different sweet cherry juice formulations were identified. PCA of GC–IMS data and LDA of E-nose results revealed that the aroma profile in LRFCJ was substantially different from that of the others. Furthermore, there was strong correlation between the E-nose and GC–IMS results. Overall, our study provides the first data on the aroma profile of sweet cherry juice according to the correlation and integration of E-nose and GC–IMS data. The results highlight the significant diversity in the aroma of fermented sweet cherry juice and confirm the potential applicability of both E-nose and GC–IMS in studying the industrial applications of fruit starter cultures.

## Data availability statement

The original contributions presented in the study are included in the article/Supplementary material, further inquiries can be directed to the corresponding author.

## Author contributions

JW: investigation, conceptualization, writing (original draft), and funding acquisition. YZ and XW: investigation, conceptualization, and validation. Y-JG: writing (review and editing). B-CW: software and formal analysis. All authors contributed to the article and approved the submitted version.

## Funding

This study was supported by the University Natural Sciences Research Project of Anhui Province (KJ2021A1009), the Hefei University Scientific Research and Development Fund (20ZR09ZDB), and the talent fund of Hefei University (20RC48).

## Conflict of interest

The authors declare that the research was conducted in the absence of any commercial or financial relationships that could be construed as a potential conflict of interest.

## Publisher’s note

All claims expressed in this article are solely those of the authors and do not necessarily represent those of their affiliated organizations, or those of the publisher, the editors and the reviewers. Any product that may be evaluated in this article, or claim that may be made by its manufacturer, is not guaranteed or endorsed by the publisher.
