# Supplementary material for: Aroma profiles of sweet cherry juice fermented by different lactic acid bacteria determined through integrated analysis of electronic nose and gas chromatography–ion mobility spectrometry
Source: Front Microbiol. 2023 Jan 16;14:1113594. doi: 10.3389/fmicb.2023.1113594 (PMC9886094; doi:10.3389/fmicb.2023.1113594)
Supplement: Supplementary file 1 [file Table_1.DOCX]

Supplementary Material

**Aroma Profiles of** **Sweet Cherry Juice Fermented by Different Lactic Acid Bacteria Determined Through Integrated Analysis of Electronic Nose and Gas Chromatography–Ion Mobility Spectrometry**

**Jun Wang ^1,2*^, Bo-Cheng Wei^1,2^, Xin Wang^1,2^, Yan Zhang^1^, Yun-Jin Gong^1^**

*** Correspondence:** Jun Wang: junw@hfuu.edu.cn

# Supplementary Table 1

**Table S1.** The peak volumes of identified volatile compounds in unfermented sweet cherry juice and sweet cherry juice inoculated with different LAB strains by gas chromatography–ion mobility spectrometry (GC–IMS).

| Compound | CJ | LAFCJ | LPFCJ | LRFCJ | Odor Description |
| --- | --- | --- | --- | --- | --- |
| acids |  |  |  |  |  |
| butanoic acid | 214.12±59.70^a^ | 171.19±8.08^a^ | 163.10±4.65^ab^ | 105.76±8.19^b^ | cheesy, buttery, fruity |
| alcohols |  |  |  |  |  |
| Linalooloxide (pyranoid) | 44.63±14.50^b^ | 37.19±2.75^b^ | 78.75±5.38^a^ | 52.96±14.21^b^ | floral honey |
| 3-Methyl-3-buten-1-ol-M | 357.61±17.16^b^ | 350.14±9.89^b^ | 410.76±21.57^a^ | 187.99±17.05^c^ | sweet fruity |
| 3-Methyl-3-buten-1-ol-D | 98.08±5.70^b^ | 93.67±1.91^b^ | 117.74±8.50^a^ | 52.96±2.95^c^ | sweet fruity |
| n-Hexanol-M | 73.95±1.91^c^ | 283.09±11.20^a^ | 277.41±7.36^a^ | 143.91±11.74^b^ | fruity, sweet, green |
| 1-butanol | 54.34±1.12^c^ | 102.74±5.53^a^ | 87.98±2.71^b^ | 105.66±5.38^a^ | balsamic, whiskey |
| 2-butanol | 391.51±7.74^b^ | 412.11±4.07^a^ | 404.99±11.74^ab^ | 353.73±7.93^c^ | sweet, apricot |
| tert-butanol | 1936.38±23.91^a^ | 947.25±71.18^c^ | 999.71±55.42^c^ | 1270.98±92.65^b^ | camphor |
| ethanol | 861.44±44.75^a^ | 744.96±29.05^b^ | 731.71±14.71^b^ | 775.64±24.99^b^ | alcoholic |
| pentan-1-ol | 20.28±4.83^a^ | 13.03±1.55^b^ | 10.92±0.63^b^ | 8.83±0.58^b^ | balsamic, bready |
| n-Hexanol-D | 10.15±2.93^b^ | 31.37±3.75^a^ | 29.15±5.27^a^ | 9.08±2.52^b^ | fruity, sweet, green |
| 2-methyl-1-propanol | 26.85±3.80^b^ | 28.95±0.85^b^ | 29.86±2.22^b^ | 52.29±3.19^a^ | ethereal, winey |
| 1-propanol | 39.31±6.14^bc^ | 69.51±5.92^a^ | 48.98±6.17^b^ | 36.37±6.57^c^ | slightly sweet, fruity |
| aldehydes |  |  |  |  |  |
| Nonanal-M | 1285.97±73.76^a^ | 594.22±40.49^c^ | 846.96±47.07^b^ | 204.15±5.76^d^ | citrus, rose, lemon peel |
| Nonanal-D | 223.05±25.53^a^ | 65.57±7.58^c^ | 94.03±6.46^b^ | 49.89±5.99^c^ | citrus, rose, lemon peel |
| Benzaldehyde-M | 656.01±15.29^a^ | 75.66±11.65^b^ | 79.17±2.64^b^ | 68.71±3.98^b^ | almond, fruity |
| Benzaldehyde-D | 185.52±9.56^a^ | 32.70±2.81^b^ | 32.81±7.71^b^ | 31.81±4.28^b^ | almond, fruity |
| (E)-2-hexenal-M | 2389.89±45.85^a^ | 451.74±52.73^c^ | 474.79±5.39^bc^ | 532.41±13.93^b^ | green, clean, fruity |
| (E)-2-hexenal-D | 6150.97±298.93^a^ | 225.95±12.96^b^ | 223.17±9.32^b^ | 241.54±11.52^b^ | green, clean, fruity |
| Hexanal-D | 3927.69±180.06^a^ | 91.24±7.51^b^ | 83.08±7.11^b^ | 76.34±2.87^b^ | green, fruity |
| Hexanal-M | 1132.79±18.34^a^ | 336.78±38.72^b^ | 299.78±12.08^b^ | 226.83±12.17^c^ | green, fruity |
| Heptanal | 144.09±5.50^b^ | 129.33±5.64^c^ | 163.57±7.86^a^ | 60.45±7.00^d^ | fresh, green, cognac |
| 3-Methyl-2-butenal | 32.93±0.93^d^ | 72.02±6.20^b^ | 89.52±5.05^a^ | 57.13±3.96^c^ | sweet, fruity, pungent |
| Octanal | 134.78±5.32^a^ | 83.36±4.20^c^ | 96.64±4.13^b^ | 62.52±7.42^d^ | citrus, green, peely |
| 2-methylbutanal-M | 132.84±9.72^a^ | 42.95±1.07^c^ | 40.41±0.29^c^ | 67.20±2.88^b^ | cocoa, coffee, nutty, |
| 3-methylbutanal-M | 158.36±12.41^a^ | 99.86±6.94^c^ | 93.09±2.30^c^ | 118.44±3.67^b^ | chocolate, peach, fatty |
| 2-methylbutanal-D | 294.48±14.90^a^ | 16.92±1.73^b^ | 15.98±1.60^b^ | 16.80±2.07^b^ | cocoa, coffee, nutty, |
| 3-methylbutanal-D | 177.52±8.66^a^ | 5.62±0.69^b^ | 6.25±0.36^b^ | 7.21±1.32^b^ | chocolate, peach, fatty |
| Pentanal | 46.41±7.36^a^ | 46.33±7.68^a^ | 48.97±4.31^a^ | 28.23±1.20^b^ | bready, fruity, berry |
| esters |  |  |  |  |  |
| Ethyl Acetate-M | 152.92±14.19^c^ | 286.95±16.76^b^ | 305.39±7.18^ab^ | 319.07±11.21^a^ | sweet, grape, rummy |
| Ethyl Acetate-D | 1365.07±43.71^c^ | 1708.61±50.98^b^ | 1759.32±56.50^ab^ | 1852.35±60.28^a^ | sweet, grape, rummy |
| Butyl acetate | 27.69±2.27^b^ | 38.67±3.86^a^ | 35.13±1.28^a^ | 29.15±1.96^b^ | fruity, banana |
| ketones |  |  |  |  |  |
| 2-nonanone-M | 46.96±8.88^c^ | 275.23±76.77^b^ | 284.76±3.07^b^ | 1024.77±35.83^a^ | cheesy, green, coconut |
| 2-nonanone-D | 24.38±3.50^b^ | 24.07±4.32^b^ | 25.26±2.94^b^ | 139.69±15.32^a^ | cheesy, green, coconut |
| 2-heptanone-M | 214.33±9.77^d^ | 1812.69±186.99^b^ | 1238.34±43.77^c^ | 2334.47±20.89^a^ | cheesy, green, creamy |
| 2-heptanone-D | 77.99±2.32^d^ | 1779.17±435.69^b^ | 875.54±75.78^c^ | 2883.69±21.25^a^ | cheesy, green, creamy |
| 3-hydroxybutan-2-one | 325.31±64.75^c^ | 865.90±22.84^b^ | 906.45±143.87^b^ | 4116.80±346.38^a^ | sweet, buttery, creamy |
| 2-Pentanone-M | 258.59±3.75^d^ | 489.68±18.21^b^ | 396.92±19.43^c^ | 773.22±18.76^a^ | sweet, fruity, banana |
| 2-Pentanone-D | 268.75±14.89^d^ | 1087.71±192.29^c^ | 538.60±34.80^b^ | 3289.89±23.01^a^ | sweet, fruity, banana |
| 2,3-butanedione | 258.14±23.90^c^ | 264.88±7.72^c^ | 303. 08±20.80^b^ | 1337.17±23.55^a^ | buttery, sweet, creamy, |
| 2-butanone | 2421.72±25.70^a^ | 947.59±161.69^b^ | 864.98±108.33^b^ | 998.07±68.32^b^ | ethereal, fruity, |
| acetone | 1572.79±50.92^c^ | 2179.23±136.01^a^ | 1999.45±63.50^b^ | 1832.14±59.09^b^ | ethereal, apple, pear |
| unidentified |  |  |  |  |  |
| 1 | 115.86±9.21^d^ | 336.42±3.21^c^ | 379.71±36.59^b^ | 609.01±11.18^a^ |  |
| 2 | 184.88±46.93^c^ | 305.27±18.18^b^ | 319.52±16.25^b^ | 387.84±20.55^a^ |  |
| 3 | 234.97±4.33^a^ | 67.11±1.60^b^ | 56.39±3.34^c^ | 54.72±9.48^c^ |  |
| 4 | 215.25±1.98^b^ | 117.30±11.23^d^ | 158.96±12.49^c^ | 1102.43±28.46^a^ |  |
| 5 | 2461.01±59.96^a^ | 1790.04±133.43^c^ | 1740.01±88.40^c^ | 2208.77±74.71^b^ |  |
| 6 | 12.86±1.69^b^ | 5.12±0.36^b^ | 8.06±2.54b | 126.45±7.48^a^ |  |
| 7 | 140.14±2.83^b^ | 165.38±14.95^a^ | 151.35±6.61^ab^ | 152.78±6.79^ab^ |  |
| 8 | 138.49±1.51^a^ | 26.42±5.42^c^ | 36.02±1.85^b^ | 23.93±2.63^c^ |  |

M indicates monomer and D indicates dimer. Values in the same row with different superscript letters are significantly different (p < 0.05). Odor descriptions were cited from http://www.thegoodscentscompany.com/index.html.
